# A Cost-Minimization Analysis of Teleconsultation Versus In-Person Care for Chronic Diseases and Rehabilitation in Medically Underserved Areas of South Korea

**DOI:** 10.3390/healthcare13050445

**Published:** 2025-02-20

**Authors:** Sei-Jong Baek, Jeong-Ah Choi, Jin-Won Noh, Hyoung-Sun Jeong

**Affiliations:** 1 Department of Health Administration, Yonsei University Graduate School, Wonju 26493, Republic of Korea; seijong0727@yonsei.ac.kr (S.-J.B.); wjddkqhfk@yonsei.ac.kr (J.-A.C.); 2 Division of Health Administration, College of Software and Digital Healthcare Convergence, Yonsei University, Wonju 26493, Republic of Korea; jinwon.noh@gmail.com

**Keywords:** telemedicine, chronic disease management, medically underserved areas, societal perspective, cost-minimization analysis

## Abstract

**Background/Objectives:** Access to healthcare in medically underserved areas remains a significant challenge in South Korea due to the concentration of healthcare resources in metropolitan regions. Telemedicine offers a promising approach to mitigating this issue, yet its cost-effectiveness in these settings remains underexplored. This study aims to conduct a cost-minimization analysis comparing teleconsultation and in-person care for chronic diseases, dementia, and rehabilitation in medically underserved areas. **Methods:** In accordance with CHEERS 2022 guidelines, this study evaluates both direct and indirect costs from a societal perspective, while accounting for costs borne by various societal stakeholders. It provides evidence to guide policy decisions in regions with significant healthcare access disparities. **Results:** Using data from South Korea’s 2018 teleconsultation pilot program involving 1232 patients, the analysis revealed that teleconsultation reduced societal costs per consultation by USD 7.92 for chronic diseases, USD 27.30 for dementia care, and USD 9.61 for rehabilitation. These savings were primarily attributed to reductions in productivity losses and transportation expenses. Furthermore, the analysis identified a shift in financial burden from patients and caregivers to government and public expenditures. **Conclusions:** The findings highlight teleconsultation’s effectiveness in reducing healthcare costs while consistently benefiting diverse patient groups, underscoring the necessity of integrating telemedicine into mainstream healthcare systems to improve access and alleviate financial strain on patients and their families.

## 1. Introduction

Essential medical resources are often inadequate in rural regions, particularly in geographically challenging areas such as islands and mountainous terrain [1,2]. Regions with low population densities often face structural limitations in healthcare resource allocation, particularly in terms of infrastructure such as healthcare facilities, due to the limited availability and variability [3]. In countries like South Korea, where medical services are primarily delivered by the private sector [4,5], the allocation of medical resources to sparsely populated regions depends on public funding and remains inherently constrained. The aging population in rural, medically underserved areas highlights the urgent necessity for adaptable strategies to enhance access to healthcare services [6].

Telemedicine has emerged as an effective approach to overcoming barriers to healthcare accessibility, driven by technological advancements [7,8]. Previous studies underscore its potential to enhance treatment efficiency and reduce healthcare costs [9,10,11,12], particularly in the management of chronic diseases such as type 2 diabetes and chronic obstructive pulmonary disease (COPD). For instance, Zakaria et al. (2024) reported that a hybrid model combining remote monitoring with in-person care resulted in a substantial reduction in HbA1c levels by 2.19% among patients with type 2 diabetes [13]. Additionally, a telemedicine intervention targeting individuals with chronic illnesses demonstrated a 35% improvement in healthcare service accessibility and a 50% decrease in indirect costs, underscoring its broader utility in addressing healthcare disparities [14].

In Korea, rapid industrialization has driven significant population and resource concentration in metropolitan areas, creating marked regional disparities in healthcare resources. Approximately half of the physicians are concentrated in metropolitan regions, whereas rural areas have access to less than 15% of the medical institutions available in urban settings, leaving these regions critically underserved [15]. To address these challenges, the Korean government initiated a teleconsultation pilot program in 1988 to provide medical services to residents in some areas with limited access to healthcare facilities. Teleconsultation involves remote medical procedures, including diagnostics and prescriptions, in collaboration with off-site physicians via telecommunication technologies without direct patient contact. The service is designed for patients with chronic diseases, dementia, or rehabilitation needs who have received an initial face-to-face consultation and are deemed suitable for teleconsultation follow-up [16].

An evaluation of the program conducted in 2015 demonstrated clinical efficacy, with hypertension patients achieving a 3.23 mmHg reduction in systolic blood pressure (from 131.32 mmHg to 128.09 mmHg) and diabetes patients showing a 0.31 percentage-point decrease in glycated hemoglobin (from 7.08% to 6.77%) within three months. A satisfaction survey conducted among 243 residents of remote island regions and 70 individuals in long-term care facilities reported high approval ratings of 83% and 87.9%, respectively, both surpassing the 77% satisfaction rate observed in the previous pilot program. Additionally, medication adherence scores, assessed on a six-point scale, showed a statistically significant improvement, increasing from 4.83 before the intervention to 5.1 afterward, suggesting that telemedicine may also contribute to long-term clinical effects [17].

The clinical efficacy of telemedicine has been substantiated in various case studies, including those conducted in South Korea [17,18,19,20]. However, systematic assessments of its economic feasibility remain insufficient. This is largely due to a lack of robust economic evaluation studies, biases associated with specific perspectives, particularly those of health systems and providers, and the omission of cost data in analyses, limiting the generalizability of the results [10,21]. These limitations constrain the broader applicability of findings and underscore the need for robust evidence to validate telemedicine as a viable solution for regions with limited medical resources.

Building on the established clinical benefits demonstrated in the preceding telemedicine pilot program, this study assumes that teleconsultation yields outcomes that are at least comparable, if not superior, to those of conventional in-person care and primarily evaluates its economic feasibility. To this end, a cost-minimization analysis is conducted to compare teleconsultation with conventional care for chronic diseases, dementia, and rehabilitation in medically underserved areas of South Korea. Adopting a societal perspective, the analysis incorporates both direct and indirect costs borne by patients, caregivers, and the government or insurer. This study evaluates telemedicine’s potential as a cost-effective alternative, particularly in regions facing significant healthcare access challenges. The findings offer valuable insights for policymakers to facilitate the integration of telemedicine into mainstream healthcare systems.

## 2. Materials and Methods

### 2.1. Study Design

This study aims to evaluate the economic efficiency of teleconsultation services compared to traditional in-person care for patients residing in medically underserved areas, from a societal perspective [22]. Building on previous findings that telemedicine achieves comparable or superior outcomes in managing chronic diseases, dementia, and rehabilitation, this study conducted a cost-minimization analysis [10,23,24]. The economic evaluation followed the CHEERS 2022 guidelines for the assessments [25].

### 2.2. Data Description

This study utilized six months of survey data collected during the 2018 pilot teleconsultation program for underserved areas, along with its evaluation program carried out by the Ministry of Health and Welfare [16,26]. The dataset comprised patients’ demographic characteristics, health profile, and information gathered through surveys (e.g., transportation costs, out-of-pocket expenses). The data provided to the research team were secondary and anonymized, containing no personally identifiable information. Access was restricted solely to the researchers, and the results were presented in aggregate form only. Public healthcare costs, telemedicine equipment procurement costs, and the opportunity costs of patients and their companions were estimated using national statistics and public documents.

The study was conducted using data from 1232 respondents out of 2396 individuals included in the performance evaluation. Among the respondents, 836 patients utilized teleconsultation services, while 396 patients received comparable in-person care. The majority of patients were female (69.6%). Age distribution was as follows: 5.7% were under 60 years, 15.7% were in their 60s, 38.9% were in their 70s, and 39.8% were 80 years or older, indicating a predominantly elderly population. Patients were divided into three groups based on their medical needs: (1) for chronic disease patients (e.g., hypertension, diabetes), 386 received teleconsultation, and 239 received in-person care; (2) for dementia patients, 370 received teleconsultation, and 110 received in-person care; (3) for rehabilitation patients, 80 received teleconsultation, and 47 received in-person care.

Medically underserved areas are typically suburban regions with a higher proportion of elderly individuals [27]. Additionally, patients suffering from chronic diseases and dementia in these areas are likely to be elderly as well [28]. This structural feature is reflected in the composition of our study sample. To verify the representativeness of our sample, we compared its gender distribution to that of the overall teleconsultation pilot program. Kim et al. (2023) reported that from 2017 to September 2023, 10,407 individuals participated, with 69% being women. The majority were elderly, aligning with our dataset. This consistency indicates that our sample reliably reflects the broader teleconsultation population [29].

The 2018 dataset is the most recent that includes both a teleconsultation group and a control group of in-person care recipients, allowing for a comprehensive evaluation of direct and indirect costs. The structural and operational framework of the 2018 pilot program remains largely consistent with contemporary teleconsultation models, apart from minor refinements such as the expansion of participating municipalities and healthcare facilities. However, for accurate cost comparisons, it is important to consider inflation by taking into account price fluctuations since 2018. Details regarding the teleconsultation model are described in the subsequent section.

### 2.3. Pilot Model of Telemedicine in Korea

Under the Medical Service Act, medical practices are required to be conducted within healthcare institutions, which implies that physicians and patients must be physically present in the same location. However, the Act allows for a form of telemedicine where physicians utilize information and communication technology (ICT) to provide medical knowledge or technical support to other healthcare professionals in remote locations. Telemedicine in Korea is restricted to collaborative care between off-site physicians and on-site healthcare professionals. As of now, direct physician-to-patient teleconsultations are not permitted in principle [16,30,31].

The telemedicine pilot program targets medically underserved regions and operates through collaboration between off-site physicians and on-site healthcare professionals (e.g., physicians, nurses) or healthcare staff. Patients residing in medically underserved areas visit nearby health centers equipped with telemedicine facilities, where off-site physicians provide diagnoses and prescriptions through on-site healthcare professionals. Off-site physicians bear the same level of legal responsibility as they would in in-person consultations. However, when the on-site healthcare professional is also a physician, the primary liability for patient care rests with the local physician unless clear evidence of malpractice by the off-site physician is established [16,30].

### 2.4. Cost Estimation for Teleconsultation and In-Person Care

The costs of teleconsultation and comparable in-person care were evaluated by categorizing expenses as direct and indirect costs. Direct costs included all expenses related to consultation and prescriptions (public and patient out-of-pocket health expenditures), along with transportation expenses for patients and their companions. In this study, transportation costs incurred by patients and caregivers for medical visits were classified as non-medical direct costs, following the approach of Kongpakwattana et al. (2020) [32]. For teleconsultation, additional costs associated with the procurement of tele-equipment were considered. Indirect costs were estimated using the Human Capital Approach (HCA), which quantifies lost working hours by translating them into monetary values based on hourly wages [33,34]. In this study, both patient and caregiver productivity losses were monetized to reflect their economic impact [35]. The formula for calculating productivity losses is as follows:

$$\begin{array}{c} Productivity\;Losses\;=\;(Patient\;hours\;\times \;Hourly\;wage\;for\;Patient)\;\\ \;+(Companion\;hours\;\times \;Hourly\;wage\;for\;companion). \end{array}$$

The following factors were taken into consideration for calculating productivity losses:

i. Travel time: Calculated using data on the average access distance to general hospitals, hospitals, clinics, and public health centers and clinics in the pilot program regions. An average speed of 40 km/h, derived from the design standards commonly applied to roads in mountainous and island areas, was used in the calculations.
ii. Consultation time: Calculated by combining the average outpatient waiting time (17.1 min) and consultation time (12.4 min), totaling approximately 30 min. For rehabilitation patients, consultation was assumed to include rehabilitation therapy, with a total duration of 1 h.
iii. Wages for patients and companions: Based on the total hourly wages of skilled agricultural, forestry, and fishery workers (USD 16.85).

All costs were expressed in USD, standardized using purchasing power parity (PPP) adjustments as of 2018. It should be noted that some cost estimates were based on authoritative sources, such as government databases; however, certain limitations may apply to these estimates. Table 1 provides data sources, and their descriptions associated with estimated costs for teleconsultation and in-person care. As this analysis was conducted from a societal perspective, costs incurred were classified into three stakeholder groups: patients, caregivers, and government/insurers. The classification criteria and associated results are outlined in the following section.

### 2.5. Validation of Robustness

To determine the principal contributors to incremental costs and assess the robustness of the cost-minimization analysis, we implemented a two-step post hoc evaluation. Initially, a univariate sensitivity analysis was performed to evaluate the isolated impact of each variable including transportation expenses, total healthcare costs, telemedicine equipment installation and maintenance fees, and productivity losses. The findings are illustrated through tornado diagrams to identify the variables exerting the greatest influence on incremental costs. Subsequently, scenario analysis was conducted to examine the stability of the outcomes under varying conditions. Guided by prior studies [36,37], the variables associated with teleconsultation and in-person care were varied within a range of ±20% to simulate realistic variations observed in practice.

## 3. Results

### 3.1. Cost Measurement

Table 1 and Table 2 summarize the societal costs associated with teleconsultation and in-person care for each disease. For chronic disease management, the estimated societal cost per teleconsultation was USD 103.88. This includes direct costs of USD 80.56, with public health expenditures (USD 40.28) being the largest component, followed by out-of-pocket expenses (USD 21.45), tele-equipment installation and maintenance (USD 13.69), and transportation costs (USD 5.15). Indirect costs, which represent productivity losses, were estimated at USD 23.32. By contrast, the estimated societal cost per in-person consultation was USD 111.80, with direct costs totaling USD 66.28. Among these, out-of-pocket expenses were the largest component (USD 38.85), followed by transportation costs (USD 15.24) and public health expenditures (USD 14.20). Indirect costs amounted to USD 45.51.

For dementia care, the estimated societal cost per teleconsultation was USD 101.76. Direct costs accounted for USD 89.12, with public health expenditures (USD 42.01) being the highest, followed by out-of-pocket expenses (USD 28.36), tele-equipment installation and maintenance (USD 13.69), and transportation costs (USD 5.07). Indirect costs were calculated at USD 12.64. In-person care for dementia had an estimated societal cost of USD 129.06 per consultation. Direct costs, totaling USD 99.12, were highest for out-of-pocket expenses (USD 55.95), followed by transportation costs (USD 28.98) and public health expenditures (USD 14.20). Indirect costs were USD 29.94.

**Figure 1:**
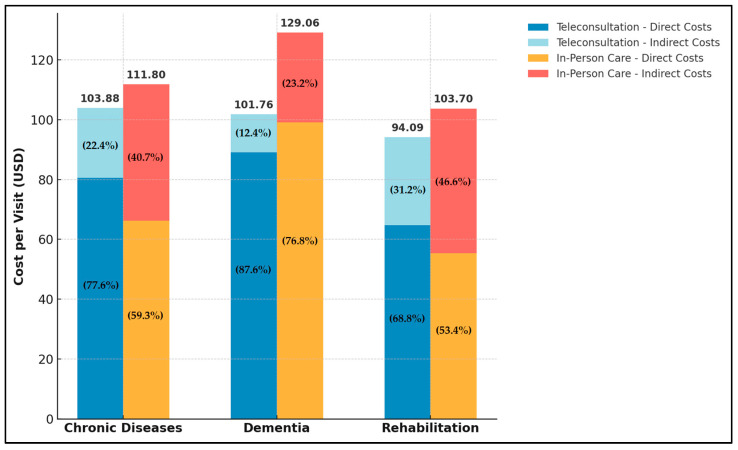
Comparison of Direct and Indirect Costs in Teleconsultation vs. In-Person Care.

For rehabilitation care, the societal cost per teleconsultation was estimated at USD 94.09. Direct costs were USD 64.73, with public health expenditures (USD 49.29) being the largest component, followed by tele-equipment installation and maintenance (USD 13.69) and transportation costs (USD 1.75). Indirect costs, reflecting productivity losses, amounted to USD 29.35. For in-person care, the societal cost per consultation was estimated at USD 103.70. Direct costs totaled USD 55.34, with out-of-pocket expenses (USD 25.09) as the highest component, followed by transportation costs (USD 16.05) and public health expenditures (USD 14.20). Indirect costs were calculated at USD 48.36. Additionally, Figure 1 illustrates a comparative analysis of the direct and indirect costs incurred in teleconsultation and in-person care for chronic diseases, dementia, and rehabilitation.

**Figure 2:**
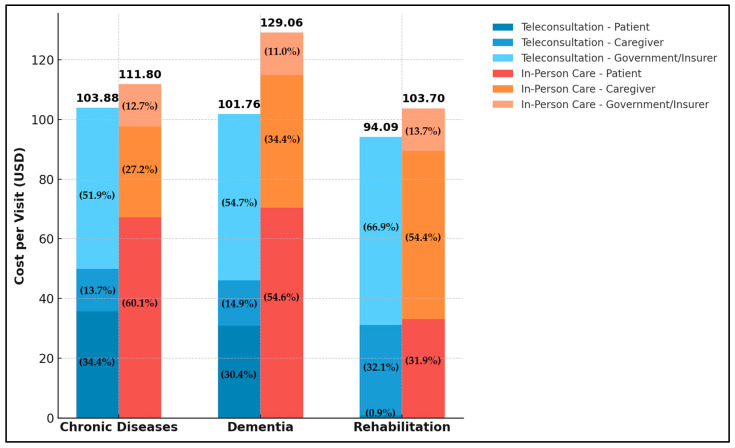
Distribution of Costs by Stakeholders Across Teleconsultation and In-Person Care.

As shown in Table 3 and Figure 2, categorizing costs by stakeholder reveals that teleconsultation results in significant cost savings for patients and caregivers across all disease areas, including chronic disease management, dementia, and rehabilitation. Specifically, patients experienced cost reductions of USD 31.54, USD 39.55, and USD 32.24 for chronic diseases, dementia, and rehabilitation, respectively, while caregivers saw reductions of USD 16.15, USD 29.25, and USD 26.16 for the same conditions. In contrast, the government and insurers faced increased costs, with a rise of USD 39.77, USD 41.50, and USD 48.78 for each of the respective disease categories. This increase primarily reflects the costs associated with telemedicine infrastructure, particularly tele-equipment procurement and maintenance. These findings suggest that although teleconsultation substantially reduces costs for patients and caregivers, the financial burden is redistributed to the government and insurers. Nevertheless, from a societal perspective, cost feasibility is ultimately sustained across all three disease categories—chronic diseases, dementia, and rehabilitation.

### 3.2. Cost-Minimization Analysis

As a result of the cost-minimization analysis, the per-visit cost of teleconsultation for chronic disease management was USD 103.88, while the cost of in-person care was USD 111.80. Under the assumption that both services provide equivalent diagnostic and prescribing capabilities, teleconsultation resulted in savings of USD 7.92 per visit.

For dementia care, the cost of teleconsultation was USD 101.76, while the cost of in-person care was USD 129.06, resulting in savings of USD 27.30 per visit. In rehabilitation care, teleconsultation was USD 94.09, while in-person care was USD 103.70, resulting in savings of USD 9.61 per visit. Across all three patient groups (chronic diseases, dementia, and rehabilitation), teleconsultation was a more cost-effective alternative than in-person care and contributed significantly to reducing societal costs.

### 3.3. Post Hoc Analysis

**Figure 3:**
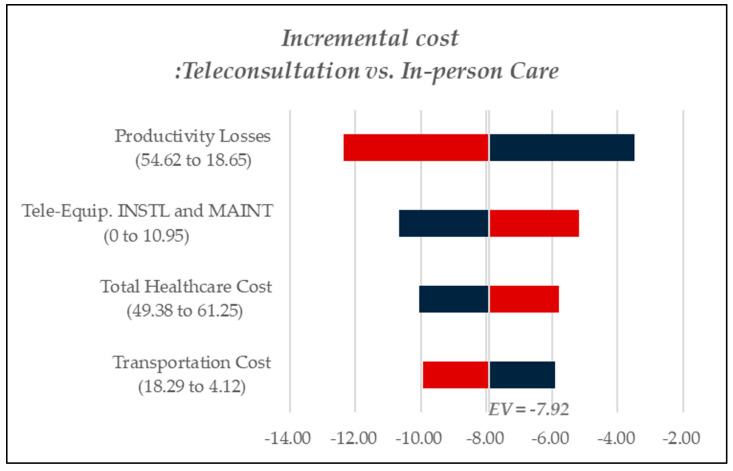
Tornado diagram for sensitivity analysis, chronic diseases.

**Figure 4:**
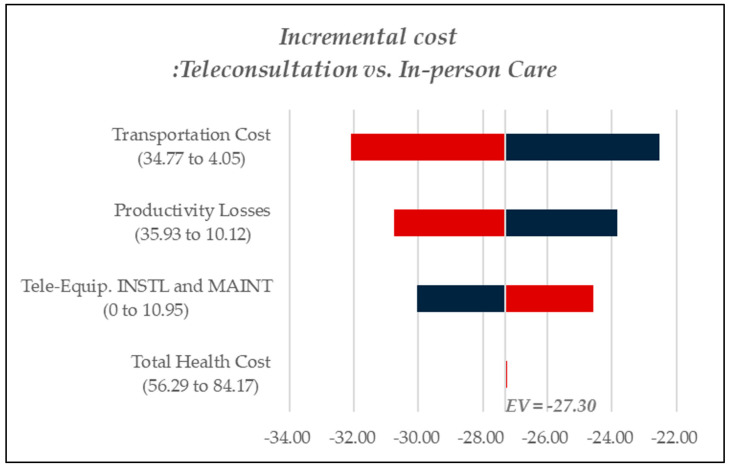
Tornado diagram for sensitivity analysis, dementia.

**Figure 5:**
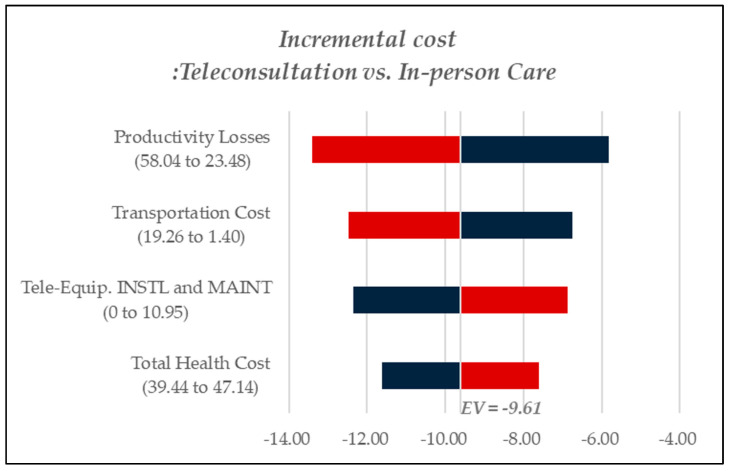
Tornado diagram for sensitivity analysis, rehabilitation.

A one-way deterministic sensitivity analysis was performed to determine the key cost drivers contributing to incremental costs in each scenario. Figure 3 reports findings for chronic disease management, Figure 4 examines cost dynamics in dementia care, and Figure 5 presents results related to rehabilitation services. In Figure 3, Figure 4 and Figure 5, red bars indicate cost increases in teleconsultation compared to in-person care, while blue bars represent cost savings. In Figure 3, teleconsultation led to a reduction in productivity losses and transportation costs (blue) but was associated with increased expenditures for teleconsultation equipment installation and overall healthcare costs (red). Likewise, in Figure 5, which pertains to rehabilitation care, productivity losses emerged as the most critical cost component. By contrast, Figure 4, focusing on dementia care, identified transportation costs as the primary cost driver, followed by productivity losses.

To assess the robustness of the findings, a scenario analysis was performed for each disease category. Key cost-driving variables, which were identified as having the greatest impact in the preceding tornado diagram analysis, were varied within a range of ±20%, with adjustments applied to both teleconsultation and in-person care costs. The resulting changes in incremental cost were analyzed (Table 4, Table 5 and Table 6). Across most tested conditions, incremental cost values were consistently negative, favoring teleconsultation. Additionally, adjustments to other variables influencing each case further supported the cost-effectiveness of teleconsultation compared to in-person care, further validating the findings of the cost-minimization analysis.

## 4. Discussion

### 4.1. Findings

This study aimed to assess the economic advantages of telemedicine compared to in-person care in regions with limited access to medical services. The findings demonstrate that teleconsultation, from a societal perspective, resulted in cost savings of USD 7.92 per visit for chronic disease management, USD 27.30 for dementia care, and USD 9.61 for rehabilitation treatment in the respective patient groups. These findings are consistent with prior research indicating that telemedicine improves patient access to care and reduces healthcare costs, particularly in chronic disease management [38,39,40]. Furthermore, they suggest that similar cost-saving effects may be observed in other healthcare systems or national contexts. The sensitivity analysis revealed that reductions in indirect costs, particularly productivity losses, along with non-medical direct costs such as transportation expenses, were the primary drivers of cost savings. These findings align with previous studies and emphasize that the primary economic benefits of telemedicine derive from reductions in non-medical costs, such as time-related expenses [12,41,42].

Stakeholder-specific cost analysis indicated that teleconsultation resulted in cost reductions for patients and caregivers, whereas expenditures for the government and insurers increased. This suggests that the introduction of telemedicine shifted the financial burden from patients and caregivers to the public sector. Since South Korea operates under a single-payer public insurance system [5], this outcome aligns with the primary objective of the teleconsultation pilot program, which was implemented to mitigate physical and financial barriers to healthcare access in medically underserved regions. Despite increased public expenditures, a societal perspective confirms that the total cost per consultation decreased across all patient groups, reinforcing the overall cost-saving potential of telemedicine.

### 4.2. Interpretations

Korea attained universal health coverage (UHC) in 1989; nevertheless, significant inequalities in healthcare access and unmet medical needs remain. These issues are primarily attributed to geographical disparities stemming from the concentration of healthcare resources in metropolitan areas, social inequities exacerbated by an aging population and information asymmetry, and delays in accessing timely healthcare services due to economic hardships [43,44,45,46]. To address these challenges, the Korean government has implemented various measures, including the designation of regional responsible medical institutions, the introduction of a primary care physician system for individuals with disabilities, and the establishment of a Medical Aid program targeting vulnerable populations [47,48,49]. Among these initiatives, the telemedicine pilot project has emerged as both a technological innovation and a policy measure, though its broader implementation remains constrained by regulatory limitations. Recognizing these challenges, this study examines key policy considerations for expanding telemedicine while maintaining financial viability and operational efficiency. Against this backdrop, this study offers several policy implications:

First, telemedicine represents a viable solution for improving healthcare accessibility in areas with constrained medical resources. Evidence from prior studies and pilot programs has shown that telemedicine’s clinical effectiveness is comparable to, and occasionally exceeds, that of traditional in-person care [10,11,12,17]. Furthermore, this study conducted a socioeconomic evaluation that accounted for opportunity costs and transportation expenses incurred by patients and their companions. The findings demonstrated that teleconsultation provided cost-saving benefits compared to in-person care across all three patient groups. These results underscore telemedicine’s role in reducing societal costs by minimizing patients’ travel expenses and productivity losses [50,51]. Notably, the implementation or expansion of telemedicine in large continental countries, where substantial distances separate rural and urban areas, could lead to significant social resource savings [52]. For instance, these findings can be compared to efforts aimed at closing the rural healthcare gap in countries like the United States or Australia [53,54].

Second, adjusting the fee structure for telemedicine appropriately could yield additional economic benefits [55]. While the cost-minimization analysis revealed that the direct medical costs of teleconsultation exceeded those of in-person care across all patient groups, two important considerations should be addressed. First, the data for this study were based on a 2018 pilot program, during which an adequate reimbursement framework had not yet been established. As a result, the fees for off-site physicians were calculated according to the emergency telemedicine pilot program’s fee standards. Importantly, these fees were intentionally set at double the rate of an initial in-person visit to facilitate the adoption of telemedicine services. Secondly, the insurer was responsible for separately reimbursing the costs incurred by off-site physicians and in-site medical personnels [55,56].

During the COVID-19 pandemic, the necessity and adoption of telemedicine services expanded significantly, both in Korea and globally [57,58]. In response, South Korea temporarily authorized telemedicine for COVID-19 treatment, with services reimbursed at 130% of the face-to-face consultation fee. If telemedicine is integrated into routine medical practice, current reimbursement rates are expected to be maintained [59]. This contrasts with countries such as Australia, France, Japan, the United Kingdom, and the United States, where telemedicine costs are generally comparable or lower to those of in-person consultations [60,61,62]. Given that teleconsultations can reduce time-related costs for healthcare providers while potentially enhancing overall revenue [63,64], they present economic benefits that extend beyond patient cost reductions. As telemedicine evolves to encompass direct physician-to-patient consultations and a wider range of medical services, it will be essential for the Korean government to reassess reimbursement structures in alignment with global standards to ensure both financial sustainability and optimized societal cost savings.

Lastly, it is worth noting that telemedicine holds significant cost-effective potential not only in physically constrained environments but also in more general contexts [42,65]. In the cost-minimization analysis, the most significant factor driving cost savings in chronic disease management was the reduction in productivity losses for patients and their companions. Similarly, in dementia and rehabilitation patient groups, where only the companions’ opportunity costs were considered, substantial cost reductions were achieved through decreased indirect costs. This study focused on elderly populations residing in medically underserved areas, using the average wage of agricultural workers (USD 16.85) to estimate productivity losses. Expanding this analysis to consider the average wage of all occupational groups (USD 22.84) could reveal even greater cost-reduction effects. These findings support the study by Patel KB et al. (2023), which demonstrated that telemedicine could mitigate the financial toxicity associated with cancer care [66]. Additionally, the findings from the CMS (Cost-Minimization Analysis) conducted by Mas, C. A. et al. (2021), which elucidated the cost-effectiveness and the enhancement of bed resource availability through Pediatric Tele-Home Care, further substantiate this argument [67].

However, when interpreting the results of this study in the context of different healthcare systems, it is essential to take regional and economic differences into account. In South Korea, the uniform reimbursement system has effectively reduced regional cost variations [68]; however, in nations with more decentralized or privatized healthcare financing, telemedicine costs may fluctuate significantly across regions. Therefore, when applying these findings, it is crucial to account for the distinct regional contexts and healthcare funding structures, as these factors may influence the cost-effectiveness of telemedicine across different systems.

### 4.3. Limitations

This study stands out for its use of a rigorous methodology consistent with the CHEERS 2022 guidelines [25], offering a comprehensive economic evaluation from a societal cost perspective that incorporates both direct and indirect costs. Nonetheless, it has certain limitations. First, although this study employs the most recently available dataset that includes both a teleconsultation group and a control group, the findings may not entirely capture recent shifts in telemedicine practices and healthcare cost dynamics. Second, the cost estimates are based on data collected over six months from a single pilot project conducted in Korea before an appropriate telemedicine reimburse rate was established, which may restrict the applicability of the results to other countries or settings. In particular, the health expenditure component is shaped by Korea’s single-payer reimbursement framework, which may not be directly generalizable to healthcare systems that rely on private insurance or alternative financing models. Third, key parameters, such as productivity losses and equipment costs, were derived from national averages, which might not adequately capture regional variability. Fourth, while this study primarily examines short-term cost savings, we acknowledge that telemedicine’s long-term benefits—such as enhanced chronic disease management and reduced hospitalization rates—may further improve its cost-effectiveness. Future research should integrate these aspects to provide a more holistic economic evaluation of teleconsultation.

## 5. Conclusions

This study confirmed that teleconsultation can significantly reduce societal costs compared to in-person care in medically underserved areas. Cost-saving effects were consistently observed across all patient groups, including those undergoing treatment for chronic diseases, dementia, and rehabilitation. These results underscore telemedicine’s potential to improve healthcare accessibility and reduce the financial burden on patients and their caregivers. Moreover, they offer crucial policy guidance, particularly for countries like South Korea, where telemedicine is still in the pilot phase and has not yet been fully integrated into the mainstream healthcare system.

To elevate telemedicine as a cornerstone of a sustainable healthcare system rather than limiting it to a cost-saving measure, the following initiatives are critical. First, teleconsultation fees must be calibrated to be lower than or at least equal to those for in-person care, thereby lowering financial barriers to widespread adoption. Second, the ongoing evaluation of telemedicine’s clinical effectiveness should be prioritized, accompanied by initiatives to improve its acceptance among healthcare professionals and patients through targeted educational and promotional efforts [10]. Future studies should not only investigate the long-term health impacts of telemedicine—such as its influence on chronic disease progression and hospitalization rates—but also evaluate its applicability across diverse social, economic, and geographic contexts. Such research will establish a comprehensive policy framework capable of addressing healthcare disparities in underserved regions, not only in Korea but also on a global scale.

## Figures and Tables

**Table 1:** Description of costs per visit for teleconsultation and in-person care.

Service	Category	Variable	Parameter	Description	Chronic Diseases	Dementia	Rehabilitation
					Value (USD)	Value (USD)	Value (USD)
Teleconsultation Service	Direct Cost	Total Health Expenditure	Public health expenditures	Public spending on teleconsultation fees per visit ^(1)^	40.28	42.01	49.29
			Out-of-pocket expenses	Out-of-pocket expenses including medication costs per visit ^(2)^ (Patient coinsurance is coverd by public spending for the pilot program)	21.45	28.36	0.00
		Tele-equipment setup and maintenance	Tele-equipment setup and maintenance	Equipment installation and maintenance costs for teleconsultation per visit ^(3)^ (depreciated over a 5-year useful life with a residual value of zero)	13.69	13.69	13.69
		Transportation cost	Transportation cost	Round-trip transportation costs to visit public health centers or clinics ^(2)^	5.15	5.07	1.75
	Indirect Cost	Productivity losses	Patient hours	Total time required for a patient to receive teleconsultation ^(4)^ (including treatment if necessary and travel time)	41 min	41 min	41 min
			Hourly wage for patient	Skilled agricultural, forestry, and fishery workers’ hourly income based on per capita ^(5)^	16.85	-	-
			Companion hours	Total time required for a companion of patient to receive teleconsultation ^(4)^ (including treatment if necessary and travel time)	41 min	45 min	1 h 44 min
			Hourly wage for companion	Skilled agricultural, forestry, and fishery workers’ hourly income based on per capita ^(5)^	16.85	16.85	16.85
In-person care	Direct Cost	Total Health Expenditure	Public health expenditures	Public spending on general consultation reimbursement fees per visit ^(1)^	14.20	14.20	14.20
			Out-of-pocket expenses	Out-of-pocket expenses including medication costs per visit ^(2)^	36.85	55.95	25.09
		Transportation cost	Transportation cost	Round-trip transportation costs to visit clinics, hospitals or general hospitals ^(2)^	15.24	28.98	16.05
	Indirect Cost	Productivity losses	Patient hours	Total time required for a patient to receive teleconsultation ^(4)^ (including treatment if necessary and travel time)	1 h 21 min	1 h 47 min	1 h 47 min
			Hourly wage for patient	Skilled agricultural, forestry, and fishery workers’ hourly income based on per capita ^(5)^	16.85	-	-
			Companion hours	Total time required for a companion of patient to receive teleconsultation ^(4)^ (including treatment if necessary and travel time)	1 h 21 min	1 h 47 min	1 h 47 min
			Hourly wage for companion	Skilled agricultural, forestry, and fishery workers’ hourly income based on per capita ^(5)^	16.85	16.85	16.85

^(1)^ Calculated based on 2018 Guide to the Pilot Program for Teleconsultation (MOHW); ^(2)^ Source: 2018 Survey on pilot program for teleconsultaiton (MOHW); ^(3)^ Calculated based on the specifications for tele-equipment (SSIS); ^(4)^ Calculated based on 2018 Patient Experience Assessment Report (KIHASA) and 2018 National Territorial Monitoring Report (KRIHS); ^(5)^ Source: 2018 Survey on Working Conditions by Employment Type (MOEL); *Note 1.* All the costs were expressed in USD Exchange rate: 1 PPP dollar = KRW 854.87.; *Note 2.* MOHW: Ministry of Health and Welfare, SSIS: Korea Social Security Information Service; HIRA: Health Insurance Review & Assessment Service; KIHASA: Korea Institute for Health and Social Affairs; KRIHS: Korea Research Institute for Human Settlements; MOEL: Ministry of Employment and Labor; PPP: Purchasing power parity.

**Table 2:** Estimated direct and indirect costs per visit for teleconsultation and in-person care.

		Chronic Diseases			Dementia			Rehabilitation		
		Teleconsultation (A)	In-Person Care (B)	Incremental Cost (A−B)	Teleconsultation (A)	In-Person Care (B)	Incremental Cost (A−B)	Teleconsultation (A)	In-Person Care (B)	Incremental Cost (A−B)
Direct Cost	Total Healthcare Cost	61.73	51.04	10.69	70.37	70.15	0.22	49.29	39.29	10.01
	Transportation Cost	5.15	15.24	−10.10	5.07	28.98	−23.91	1.75	16.05	−14.29
	Tele-Equipment Installation and Maintenance Cost	13.69	-	13.69	13.69	-	13.69	13.69	-	13.69
Indirect Cost	Productivity Losses	23.32	45.51	−22.20	12.64	29.94	−17.30	29.35	48.36	−19.01
Total Cost		103.88	111.80	−7.92	101.76	129.06	−27.30	94.09	103.70	−9.61

*Note.* All the costs were expressed in USD Exchange rate: 1 PPP dollar = KRW 854.87. As shown in Table 1, public health expenditures and out-of-pocket expenses are the components of total healthcare cost.

**Table 3:** Estimated costs per visit by stakeholder group: teleconsultation vs. in-person care.

	Chronic Diseases			Dementia			Rehabilitation		
	Teleconsultation (A)	In-Person Care (B)	Incremental Cost (A−B)	Teleconsultation (A)	In-Person Care (B)	Incremental Cost (A−B)	Teleconsultation (A)	In-Person Care (B)	Incremental Cost (A−B)
Patient-Borne Costs	35.68	67.23	−31.54	30.89	70.44	−39.55	0.88	33.12	−32.24
Caregiver Burden (Economic and Time Costs)	14.23	30.38	−16.15	15.18	44.43	−29.25	30.23	56.39	−26.16
Government and Public Expenditures	53.96	14.20	39.77	55.70	14.20	41.50	62.98	14.20	48.78
Total Cost	103.88	111.80	−7.92	101.76	129.06	−27.30	94.09	103.70	−9.61

*Note.* All the costs were expressed in USD Exchange rate: 1 PPP dollar = KRW 854.87.

**Table 4:** Scenario analysis of incremental costs for chronic diseases.

Change in Transportation Cost	Teleconsultation (A)	In-Person Care (B)	Incremental Cost (A-B)	Result
A: −20% B: +20%	99.21	120.90	−21.69	Negative (−)
A: −20% B: Unchanged	99.21	111.80	−12.59	Negative (−)
A: −20% B: −20%	99.21	102.70	−3.48	Negative (−)
A: Unchanged B: +20%	103.88	120.90	−17.03	Negative (−)
Baseline	103.88	111.80	−7.92	Negative (−)
A: Unchanged B: −20%	103.88	102.70	1.18	Positive (+)
A: +20% B: +20%	108.54	120.90	−12.36	Negative (−)
A: +20% B: Unchanged	108.54	111.80	−3.26	Negative (−)
A: +20% B: −20%	108.54	102.70	5.84	Positive (+)

**Table 5:** Scenario analysis of incremental costs for dementia.

Change in Productivity Loss	Teleconsultation (A)	In-Person Care (B)	Incremental Cost (A-B)	Result
A: −20% B: +20%	100.75	134.86	−34.11	Negative (−)
A: −20% B: Unchanged	100.75	129.06	−28.31	Negative (−)
A: −20% B: −20%	100.75	123.27	−22.52	Negative (−)
A: Unchanged B: +20%	101.76	134.86	−33.09	Negative (−)
Baseline	101.76	129.06	−27.30	Negative (−)
A: Unchanged B: −20%	101.76	123.27	−21.50	Negative (−)
A: +20% B: +20%	102.78	134.86	−32.08	Negative (−)
A: +20% B: Unchanged	102.78	129.06	−26.28	Negative (−)
A: +20% B: −20%	102.78	123.27	−20.49	Negative (−)

**Table 6:** Scenario analysis of incremental costs for rehabilitation.

Change in Productivity Losses	Teleconsultation (A)	In-Person Care (B)	Incremental Cost (A-B)	Result
A: −20% B: +20%	88.22	113.37	−25.16	Negative (−)
A: −20% B: Unchanged	88.22	103.70	−15.48	Negative (−)
A: −20% B: −20%	88.22	94.03	−5.81	Negative (−)
A: Unchanged B: +20%	94.09	113.37	−19.29	Negative (−)
Baseline	94.09	103.70	−9.61	Negative (−)
A: Unchanged B: −20%	94.09	94.03	0.06	Positive (+)
A: +20% B: +20%	99.96	113.37	−13.41	Negative (−)
A: +20% B: Unchanged	99.96	103.70	−3.74	Negative (−)
A: +20% B: −20%	99.96	94.03	5.93	Positive (+)

## Data Availability

An aggregate summary of the data generated during this study is provided within this published article. The datasets used and/or analyzed during the current study are available from the corresponding author on reasonable request.
